# Brain Magnetic Resonance Imaging Findings of Shiga Toxin-producing *Escherichia coli* Hemolytic Uremic Syndrome-associated Encephalopathy

**DOI:** 10.31662/jmaj.2024-0201

**Published:** 2024-11-11

**Authors:** Takashi Nakamura, Hirohisa Fujikawa, Norimichi Uenishi

**Affiliations:** 1 Department of Emergency and General Internal Medicine, Fujita Health University School of Medicine, Aichi, Japan; 2 Department of Internal Medicine, Suwa Central Hospital, Nagano, Japan; 3 Center for General Medicine Education, School of Medicine, Keio University, Tokyo, Japan

**Keywords:** Shiga toxin-producing *Escherichia coli*, hemolytic uremic syndrome, encephalopathy, neurological manifestation, seizure, head magnetic resonance imaging, thin-section, pons

An 18-year-old female presented to our hospital with a 3-day history of hematochezia. Laboratory examination revealed hemolytic anemia, thrombocytopenia, and acute kidney injury. Stool cultures of Shiga toxin-producing *Escherichia coli* (STEC) O157: H7 confirmed the presence of typical hemolytic uremic syndrome (HUS).

Postadmission, she developed generalized tonic-clonic seizures. Brain magnetic resonance imaging (MRI) revealed bilateral symmetrical T2-fluid-attenuated inversion recovery (FLAIR) hyperintensities in the dorsal pontine base and thalamus (Figure 1A and 1B). The lesions showed high signal at diffusion-weighted imaging and apparent diffusion coefficient maps, indicating T2-shine through (Figure 1C, 1D, 1E and 1F). Considering the clinical course and imaging findings, this case was diagnosed with STEC-HUS-associated encephalopathy. After five rounds of plasma exchange and levetiracetam administration, she remained seizure-free and MRI signal changes improved (Figure 1G, 1H, 1I, 1J, 1K and 1L).

**Figure 1.**
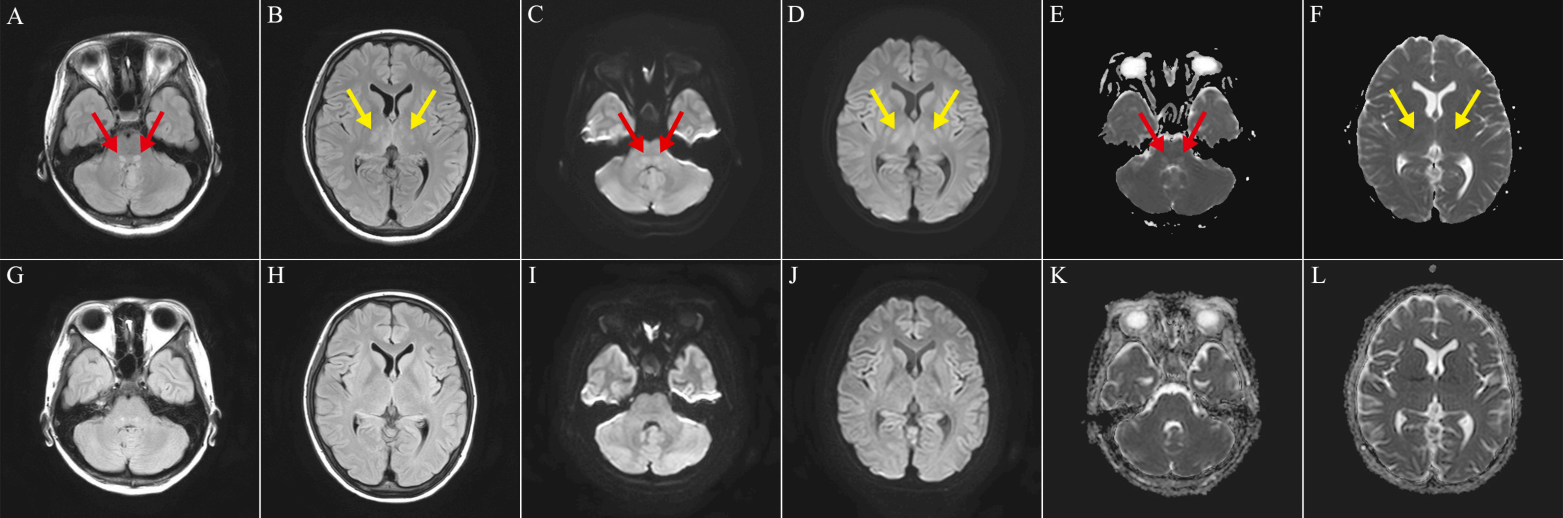
(A-F) Axial brain magnetic resonance imaging (MRI) of the patient showed bilateral symmetrical hyperintensities in the dorsal pontine base (red arrows) and thalamus (yellow arrows) on day 5 of hospitalization (A and B, T2-fluid-attenuated inversion recovery [FLAIR]; C and D, diffusion-weighted imaging; and E and F, apparent diffusion coefficient maps). (G-L) MRI signal changes in the pons and thalamus improved on day 12 of hospitalization (G and H, T2-FLAIR; I and J, diffusion-weighted imaging; and K and L, apparent diffusion coefficient maps).

Neurological manifestations of STEC-HUS are life-threatening complications, with an incidence of 17%-34%, and fatal prognostic factors ^(1), (2)^. Upon imaging, the lesions imply a nearly symmetrical distribution pattern, including bilateral basal ganglia, bilateral centrum semiovale, bilateral thalami, and bilateral brainstem ^(3)^. Among them, transient symmetric vasogenic edema of the central pons (T2-weighted or T2-FLAIR imaging) is a relatively characteristic finding ^(4)^. Transient T2 hyperintensities in the brainstem have been observed in patients with other diseases, including metronidazole encephalopathy and herpes simplex virus encephalitis ^(4)^. However, in this case, because the diagnosis of STEC-HUS was bacteriologically confirmed and no history of other diseases (e.g., no metronidazole use and no fever to support the diagnosis of herpes simplex virus encephalitis) was suggested, STEC-HUS-associated encephalopathy was diagnosed. Pontine hyperintensities are typically small and can be overlooked. Thin-section (3 mm) brain MRI may lead to an accurate diagnosis, thereby improving prognosis ^(4)^.

## Article Information

### Conflicts of Interest

None

### Author Contributions

TN acquired data and drafted the manuscript. HF and NU reviewed and supervised the manuscript.

### Informed Consent

Informed consent was obtained for this manuscript.

### Approval by Institutional Review Board (IRB)

IRB approval was not required for this study.
